# Supplementary material for: Identifying Spatial Co-occurrence in Healthy and InflAmed tissues (ISCHIA)
Source: Mol Syst Biol. 2024 Jan 15;20(2):98–119. doi: 10.1038/s44320-023-00006-5 (PMC10897385; doi:10.1038/s44320-023-00006-5)
Supplement: Supplementary file 4 — Expanded View Figures [file 44320_2023_6_MOESM4_ESM.pdf]

Expanded View Figures

Figure EV1. Composition-aware clustering and cell type co-occurrence in mouse brain Visium data.

(**A**) A Visium sample of a mouse brain coronal section (10× Genomics) is deconvoluted using a scRNAseq reference (Tasic et al, 2016), yielding 5 composition classes. Scale bar, 1 mm. (**B**) Diagonal matrix plot depicting cell type co-occurrences in every CC. Co-occurrence is positive when observed more frequently than expected ($P < 0.05$), random when there is no significant difference, negative when observed less than expected ($P < 0.05$). Underlying statistical analysis outlined in "Methods."

© The Author(s)

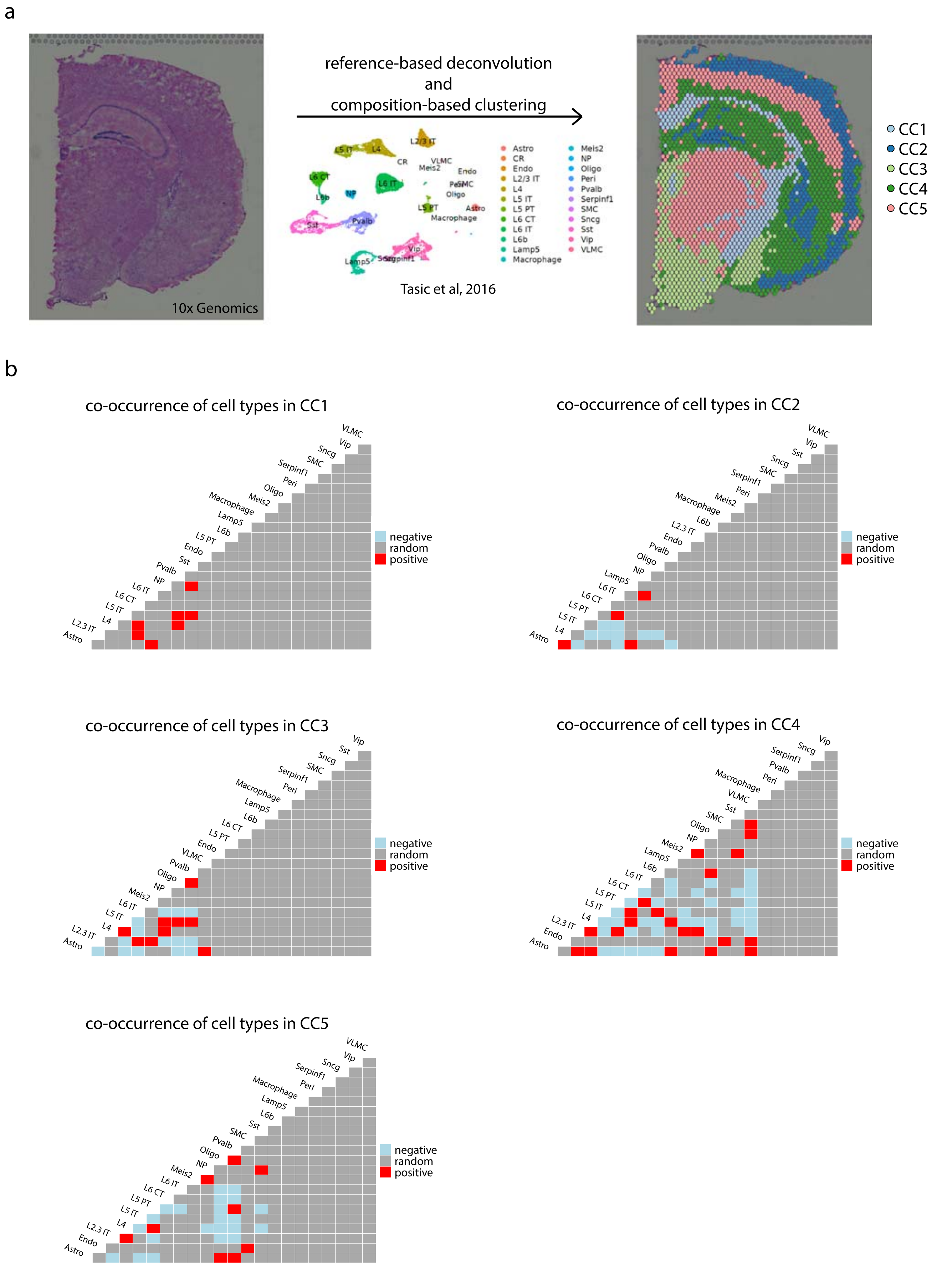
a
10x Genomics
reference-based deconvolution
and
composition-based clustering
Tasic et al, 2016
Astro
CR
Endo
L2/3 IT
L4
L5 IT
L5 PT
L6 CT
L6 IT
L6b
Lamp5
Macrophage
Meis2
NP
Oligo
Peri
Pvalb
Serpinf1
SMC
Sncg
Sst
Vip
VLMC
CC1
CC2
CC3
CC4
CC5
b
co-occurrence of cell types in CC1
co-occurrence of cell types in CC2
co-occurrence of cell types in CC3
co-occurrence of cell types in CC4
co-occurrence of cell types in CC5
negative
random
positive

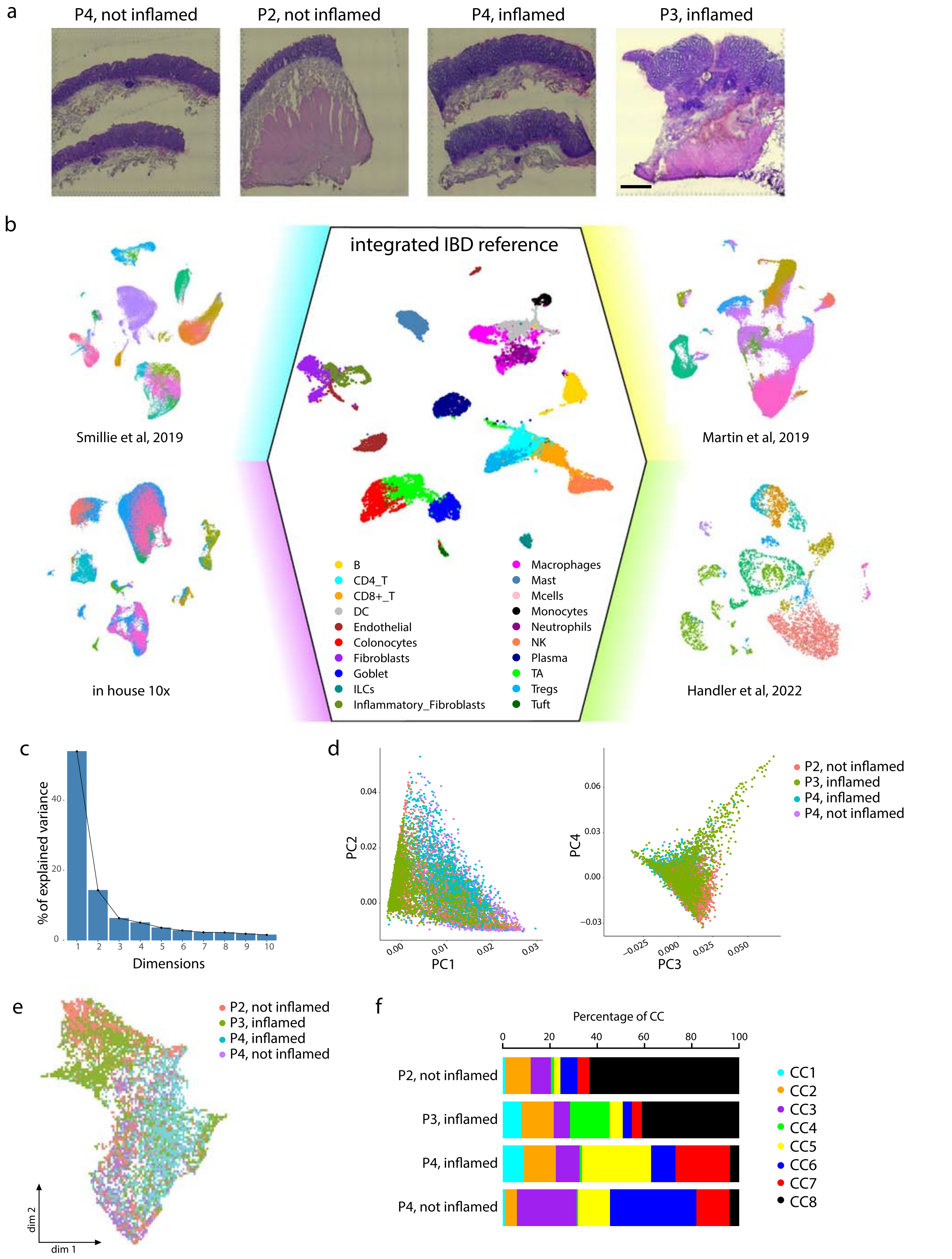
a
P4, not inflamed
P2, not inflamed
P4, inflamed
P3, inflamed
b
integrated IBD reference
Smillie et al, 2019
Martin et al, 2019
in house 10x
Handler et al, 2022
B
CD4_T
CD8+_T
DC
Endothelial
Colonocytes
Fibroblasts
Goblet
ILCs
Inflammatory_Fibroblasts
Macrophages
Mast
Mcells
Monocytes
Neutrophils
NK
Plasma
TA
Tregs
Tuft
c
% of explained variance
Dimensions
d
PC1
PC2
PC3
PC4
P2, not inflamed
P3, inflamed
P4, inflamed
P4, not inflamed
e
P2, not inflamed
P3, inflamed
P4, inflamed
P4, not inflamed
dim 1
dim 2
f
Percentage of CC
P2, not inflamed
P3, inflamed
P4, inflamed
P4, not inflamed
CC1
CC2
CC3
CC4
CC5
CC6
CC7
CC8

 © The Author(s)

Figure EV2. Composition-aware clustering of human colon Visium data.

(**A**) Hematoxylin–eosin staining of colon resections from four samples (a total of 3 ulcerative colitis patients, 2 inflamed and 2 not inflamed samples) analyzed by Visium ST (10× Genomics). Scale bar, 1 mm. (**B**) Integration of published and in house scRNAseq datasets yields a comprehensive IBD reference with a total of 51 patients. Dots represent single cells, colored by cell type. (**C**) Percent of variance of the deconvolution matrix explained by the first 10 principal components. PCs 1–4 explain more than 80% of the variance. (**D**) PC plot showing no association of any sample with a particular principal component. (**E**) Dimensionality reduction of spots colored by sample. (**F**) Percentage of CC per sample.

© The Author(s)

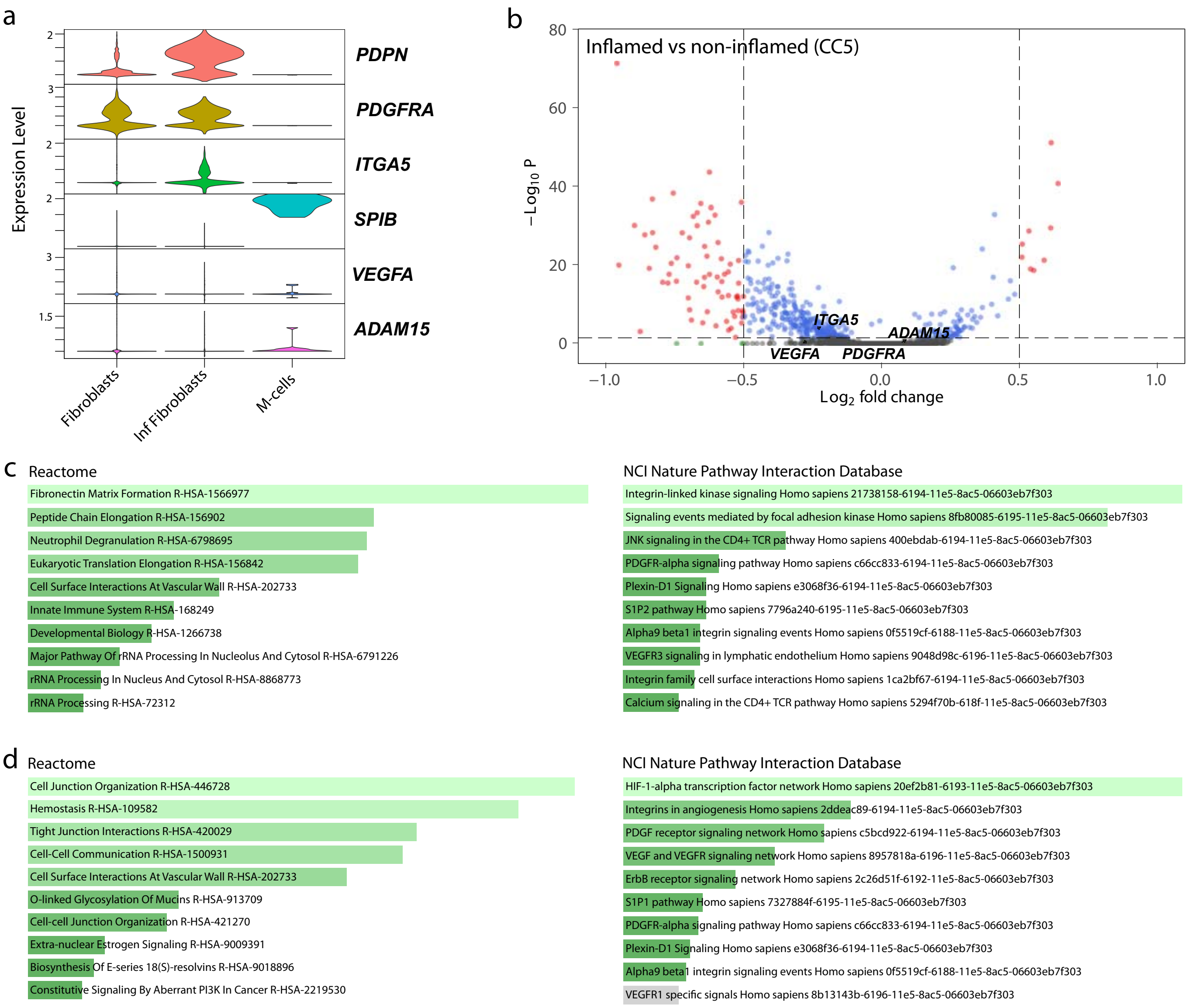

Figure EV3. Co-occurring LR pairs are associated with a transcriptional signature.

(**A**) Expression of cell type markers (*PDPN, PDGFRA, SPIB*) and LR pairs (*PDGFRA, VEGFA, ITGA5, ADAM15*) by fibroblasts and M cells in integrated IBD scRNAseq reference. (**B**) Differential gene expression within CC5, inflamed vs non-inflamed (4 samples, 3 patients). Non-parametric Wilcoxon rank sum test. (**C**) Reactome and NCI Nature Interaction Database pathway enrichment of *ITGA5-ADAM15*-associated genes. (**D**) Reactome and NCI Nature Interaction Database pathway enrichment of *VEGFA-PDGFRA*-associated genes.

 © The Author(s)

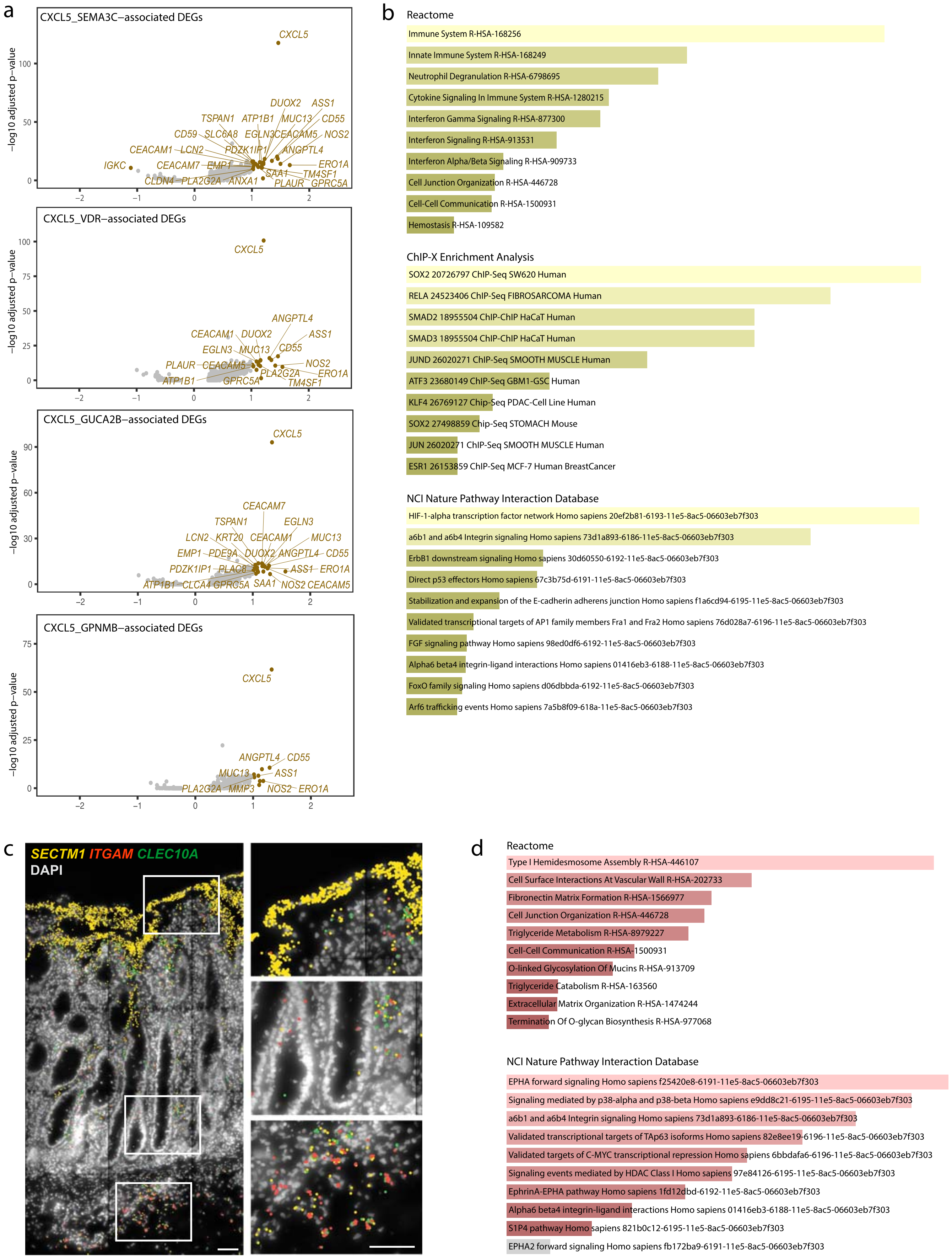
a
CXCL5_SEMA3C–associated DEGs
−log10 adjusted p−value
CXCL5
DUOX2 ASS1
TSPAN1 ATP1B1 MUC13 CD55
CD59 SLC6A8 EGLN3 CEACAM5 NOS2
CEACAM1 LCN2 PDZK1IP1 ANGPTL4
IGKC CEACAM7 EMP1 ERO1A
SAA1 TM4SF1
CLDN4 PLA2G2A ANXA1 PLAUR GPRC5A
CXCL5_VDR–associated DEGs
CXCL5
ANGPTL4
CEACAM1 DUOX2 ASS1
EGLN3 MUC13 CD55
PLAUR CEACAM5 NOS2
PLA2G2A ERO1A
ATP1B1 GPRC5A TM4SF1
CXCL5_GUCA2B–associated DEGs
CXCL5
CEACAM7
TSPAN1 EGLN3
LCN2 KRT20 CEACAM1 MUC13
EMP1 PDE9A DUOX2 ANGPTL4 CD55
PDZK1IP1 PLAC8 ASS1 ERO1A
ATP1B1 CLCA4 GPRC5A SAA1 NOS2 CEACAM5
CXCL5_GPNMB–associated DEGs
CXCL5
ANGPTL4 CD55
MUC13 ASS1
PLA2G2A MMP3 NOS2 ERO1A
b
Reactome
Immune System R-HSA-168256
Innate Immune System R-HSA-168249
Neutrophil Degranulation R-HSA-6798695
Cytokine Signaling In Immune System R-HSA-1280215
Interferon Gamma Signaling R-HSA-877300
Interferon Signaling R-HSA-913531
Interferon Alpha/Beta Signaling R-HSA-909733
Cell Junction Organization R-HSA-446728
Cell-Cell Communication R-HSA-1500931
Hemostasis R-HSA-109582
ChIP-X Enrichment Analysis
SOX2 20726797 ChIP-Seq SW620 Human
RELA 24523406 ChIP-Seq FIBROSARCOMA Human
SMAD2 18955504 ChIP-ChIP HaCaT Human
SMAD3 18955504 ChIP-ChIP HaCaT Human
JUND 26020271 ChIP-Seq SMOOTH MUSCLE Human
ATF3 23680149 ChIP-Seq GBM1-GSC Human
KLF4 26769127 Chip-Seq PDAC-Cell Line Human
SOX2 27498859 Chip-Seq STOMACH Mouse
JUN 26020271 ChIP-Seq SMOOTH MUSCLE Human
ESR1 26153859 ChIP-Seq MCF-7 Human BreastCancer
NCI Nature Pathway Interaction Database
HIF-1-alpha transcription factor network Homo sapiens 20ef2b81-6193-11e5-8ac5-06603eb7f303
a6b1 and a6b4 Integrin signaling Homo sapiens 73d1a893-6186-11e5-8ac5-06603eb7f303
ErbB1 downstream signaling Homo sapiens 30d60550-6192-11e5-8ac5-06603eb7f303
Direct p53 effectors Homo sapiens 67c3b75d-6191-11e5-8ac5-06603eb7f303
Stabilization and expansion of the E-cadherin adherens junction Homo sapiens f1a6cd94-6195-11e5-8ac5-06603eb7f303
Validated transcriptional targets of AP1 family members Fra1 and Fra2 Homo sapiens 76d028a7-6196-11e5-8ac5-06603eb7f303
FGF signaling pathway Homo sapiens 98ed0df6-6192-11e5-8ac5-06603eb7f303
Alpha6 beta4 integrin-ligand interactions Homo sapiens 01416eb3-6188-11e5-8ac5-06603eb7f303
FoxO family signaling Homo sapiens d06dbbda-6192-11e5-8ac5-06603eb7f303
Arf6 trafficking events Homo sapiens 7a5b8f09-618a-11e5-8ac5-06603eb7f303
c
SECTM1 ITGAM CLEC10A
DAPI
d
Reactome
Type I Hemidesmosome Assembly R-HSA-446107
Cell Surface Interactions At Vascular Wall R-HSA-202733
Fibronectin Matrix Formation R-HSA-1566977
Cell Junction Organization R-HSA-446728
Triglyceride Metabolism R-HSA-8979227
Cell-Cell Communication R-HSA-1500931
O-linked Glycosylation Of Mucins R-HSA-913709
Triglyceride Catabolism R-HSA-163560
Extracellular Matrix Organization R-HSA-1474244
Termination Of O-glycan Biosynthesis R-HSA-977068
NCI Nature Pathway Interaction Database
EPHA forward signaling Homo sapiens f25420e8-6191-11e5-8ac5-06603eb7f303
Signaling mediated by p38-alpha and p38-beta Homo sapiens e9dd8c21-6195-11e5-8ac5-06603eb7f303
a6b1 and a6b4 Integrin signaling Homo sapiens 73d1a893-6186-11e5-8ac5-06603eb7f303
Validated transcriptional targets of TAp63 isoforms Homo sapiens 82e8ee19-6196-11e5-8ac5-06603eb7f303
Validated targets of C-MYC transcriptional repression Homo sapiens 6bbdafa6-6196-11e5-8ac5-06603eb7f303
Signaling events mediated by HDAC Class I Homo sapiens 97e84126-6195-11e5-8ac5-06603eb7f303
EphrinA-EPHA pathway Homo sapiens 1fd12dbd-6192-11e5-8ac5-06603eb7f303
Alpha6 beta4 integrin-ligand interactions Homo sapiens 01416eb3-6188-11e5-8ac5-06603eb7f303
S1P4 pathway Homo sapiens 821b0c12-6195-11e5-8ac5-06603eb7f303
EPHA2 forward signaling Homo sapiens fb172ba9-6191-11e5-8ac5-06603eb7f303

© The Author(s)

Figure EV4. Differential co-occurrence analysis of the surfaceome reveals concerted tissue responses.

(**A**) *CXCL5*-interactions associated DEGs. Non-parametric Wilcoxon rank sum test. (**B**) Reactome, ChiP-X Enrichment Analysis and NCI Nature pathway enrichment of shared *CXCL5*-associated DEGs. (**C**) Molecular Cartography image of *SECTM1*, *ITGAM* (neutrophils) and *CLEC10A* (DCs) expression. Scale bar, 20 μm. (**D**) Reactome and NCI Nature pathway enrichment of *SECTM1*-associated DEGs.

 © The Author(s)
